# Four principles of bio-musicology

**DOI:** 10.1098/rstb.2014.0091

**Published:** 2015-03-19

**Authors:** W. Tecumseh Fitch

**Affiliations:** Department of Cognitive Biology, University of Vienna, Vienna, Austria

**Keywords:** musicality, bio-musicology, comparative approach, rhythm, dance, popular music

## Abstract

As a species-typical trait of *Homo sapiens*, musicality represents a cognitively complex and biologically grounded capacity worthy of intensive empirical investigation. Four principles are suggested here as prerequisites for a successful future discipline of bio-musicology. These involve adopting: (i) a multicomponent approach which recognizes that musicality is built upon a suite of interconnected capacities, of which none is primary; (ii) a pluralistic Tinbergian perspective that addresses and places equal weight on questions of mechanism, ontogeny, phylogeny and function; (iii) a comparative approach, which seeks and investigates animal homologues or analogues of specific components of musicality, wherever they can be found; and (iv) an ecologically motivated perspective, which recognizes the need to study widespread musical behaviours across a range of human cultures (and not focus solely on Western art music or skilled musicians). Given their pervasiveness, dance and music created for dancing should be considered central subcomponents of music, as should folk tunes, work songs, lullabies and children's songs. Although the precise breakdown of capacities required by the multicomponent approach remains open to debate, and different breakdowns may be appropriate to different purposes, I highlight four core components of human musicality—song, drumming, social synchronization and dance—as widespread and pervasive human abilities spanning across cultures, ages and levels of expertise. Each of these has interesting parallels in the animal kingdom (often analogies but in some cases apparent homologies also). Finally, I suggest that the search for universal capacities underlying human musicality, neglected for many years, should be renewed. The broad framework presented here illustrates the potential for a future discipline of bio-musicology as a rich field for interdisciplinary and comparative research.

## 1. Introduction: bio-musicology and ‘musicality’

In April 2014, I presented a short ‘position statement’ on the first day of the Lorentz Conference on Musicality (cf. the introduction to this issue by Honing *et al*. [1]). My goal was to present several principles that I believed were necessary foundations for a future discipline of bio-musicology, but that I also thought might be controversial and spark discussion. To my surprise, however, with few exceptions these proposed principles were readily accepted by the very diverse set of academics assembled at that conference. I present these principles and briefly explore some of their implications for current and future bio-musicological research in the following sections.

### (a) Defining the object of study: ‘musicality’ versus music

‘Bio-musicology’ is the biological study of musicality in all its forms. Human ‘musicality’ refers to the set of capacities and proclivities that allows our species to generate and enjoy music in all of its diverse forms. A core tenet of bio-musicology is that musicality is deeply rooted in human biology, in a form that is typical of our species and broadly shared by members of all human cultures. While music, the product of human musicality, is extremely diverse, musicality itself is a stable aspect of our biology and thus can be productively studied from comparative, neural, developmental and cognitive perspectives. The bio-musicological approach is comparative in at least two senses: first that it takes as its domain all of human music-making (not privileging any one culture, or ‘art music’ created by professionals) and second that it seeks insight into the biology of human musicality, wherever possible, by looking at related traits in other animals.

Note that there is no contradiction in seeing musicality as a universal aspect of human biology, while accepting the vast diversity of music itself, across cultures or over historical time within a culture. While the number of possible songs is unlimited, singing as an activity can be insightfully analysed using a relatively small number of parameters (Is singing done in groups or alone? With or without instrumental accompaniment? Is it rhythmically regular or not?, etc.). As Alan Lomax showed in his monumental cantometrics research programme, such a classification can provide insights into both the unity and diversity of music, as instantiated in human cultures across the globe [2–4]. Furthermore, the form and function of the vocal apparatus that produces song is shared by all normal humans, from a newborn to Pavarotti [5], and indeed the overall form and function of our vocal apparatus is shared with many other mammal species from mice to elephants [6,7].

While ethnomusicology traditionally focuses on the form and social function of songs (and other products of musicality), bio-musicology seeks an understanding of the more basic and widely shared capabilities underlying our capacity to make music, such as singing. There is no conflict between these endeavours, and indeed there is great potential for synergy among them since each can feed the other with data, hypotheses and potential generalizations.

Having thus clarified the object of study and general approach, I turn to four core principles that I believe should provide the foundations for effective, productive scientific inquiry into musicality.

## 2. Four foundational principles of bio-musicology

### (a) The ‘multicomponent’ principle: musicality encompasses multiple components

The first principle is uncontroversial among musicologists (if not always clearly recognized by biologists): productive research into musicality requires that we identify and study its multiple interacting components. This basic notion is familiar from music theory, where Western music is commonly dissected into separate components, including rhythm, melody and harmony, each considered to be important aspect of a typical piece of music. But we cannot assume that this particular traditional theoretical breakdown is the appropriate one from a biological perspective, nor that ‘rhythm’ or ‘harmony’ are themselves monolithic capacities. Rather, we should be ready to explore multiple componential frameworks open-mindedly, and allow the data to steer us to the insightful subdivisions. We should also accept that different componential breakdowns might be appropriate for different purposes. For example, from a biological, comparative perspective it is useful to seek aspects of human musicality that have parallels in other species (I explore this approach below, concluding that singing, drumming and dancing all find meaningful homologues or analogues in non-human animals). But a developmental researcher investigating the time course of musical development might find a different taxonomy appropriate, and a neuroscientist yet another. There is no one ‘true’ or ‘correct’ breakdown.

The multicomponent perspective is crucial for the biological study of musicality, for although it seems true that no non-human species possesses ‘music’ in its full human form(s), it is nonetheless equally true that many animal species share some of the capacities underlying human musicality, spanning from broadly shared capabilities like pitch and time perception, to less common abilities like synchronization or vocal learning. Indeed, based on current data, it seems likely that most of the basic capacities comprising human musicality are shared with at least some other animal species; what is unusual about humans may simply be that we combine all of these abilities. This hypothesis will be discussed further below, as will the question of meaningful possibilities for subdivision. Principle one does not entail accepting any particular taxonomy of components, but rather the general need for *some* such multicomponent viewpoint. Thus, in a nutshell, principle one exhorts us to ‘divide and conquer’.

### (b) The principle of explanatory pluralism: consider all of Tinbergen's explanatory levels

The second principle is familiar to biologists, but less so to psychologists or musicologists. The essential insight for this second principle was provided over 50 years ago by Nobel Prize winning ethologist Niko Tinbergen [8]: that any biological phenomenon can be understood, and its causation explained, at multiple different levels. Tinbergen divided these levels into two broad families: proximate and ultimate explanations. *Proximate factors* include all those that help explain why some particular organism does something, and include *mechanistic* explanations (‘How does it work?’) and *ontogenetic* or developmental explanations (‘How did it develop in this particular organism's lifetime?’). These are the domains of (neuro) physiology and developmental biology, respectively.

But, thanks to Darwin, biologists are not fully satisfied by just these two levels of explanation; we also strive to understand life from the viewpoint of the longer time scale of evolution, and to understand how and why some particular capability arose in a *species* (or group of species). This is the domain of *ultimate factors*, traditionally divided into questions about *phylogeny* (the evolutionary history of acquisition and modification of a trait) and questions concerning the ultimate *function* or ‘survival value’ of the trait (‘How does it help those that possess the trait in a population to survive and reproduce more effectively than others?’). Both of these levels are core components of modern evolutionary biology.

Tinbergen's four levels of explanation (sometimes called his ‘Four Whys’) were extremely important when he proposed them because they provided a resolution to a long-running and unproductive debate between (mostly) English-speaking scientists like Theodore Schneirla and Daniel Lehrman who focused on mechanistic and ontogenetic explanations [9], and the (mostly) continental European scientists like Konrad Lorenz and Tinbergen, who were comparative biologists interested in ultimate explanations. Tinbergen pointed out that there is actually no conflict between these different types of explanation, and that full understanding of any biological trait requires answers at *all four* levels of causation. Thus, we know that male songbirds sing in spring because their testosterone levels are high (a mechanistic explanation), but we also know that an important function of song is to defend a territory and attract mates (an ultimate, functional explanation). In this well-understood case, we know that both explanations are correct and important, and it would be a waste of time to argue that one of these factors and not the other provide the ‘true’ explanation. Tinbergen's rule—‘Attend to all levels of biological explanation!’—provides a widely accepted antidote to such unproductive debate. It is generally taught to students of biology early in their training.

Applying Tinbergen's approach to musicality yields several important insights. Mechanistic questions in the domain of musicality include issues such as ‘What are the neural bases for rhythm perception?’ (for which see Merchant *et al*. [10]) or ‘What physiological and cognitive factors underlie a skilled singer's abilities?’. Ontogenetic issues include ‘At what age do infants perceive relative pitch relationships?’ or ‘Does early exposure to musical performance enhance pitch perception?’ [11–13]. Of course, there is no hard and fast line dividing these two types of explanations, and for many (perhaps most) traits they are tightly intertwined. For example, it now seems clear that early and intensive exposure to music during ontogeny causes measurable changes in neural mechanisms later in life (e.g. [14–16]). Of Tinbergen's four main questions, these two proximate foci are currently very active research areas, and represent core empirical domains of bio-musicology.

Regarding ultimate questions, it is often thought that the core evolutionary question in bio-musicology concerns whether or not music is an adaptation (and if so, for what). Thus, for example, Steven Pinker provocatively suggested that music is simply a by-product of other cognitive abilities (a form of ‘auditory cheesecake’), and not itself an adaptation [17]. Many subsequent scholars have challenged this hypothesis with specific proposals that music is an adaptation for particular functions [18–25]. This debate is reviewed elsewhere [18,26,27] and, since I do not find it particularly productive, I will not discuss it further here. But note that Tinbergen stressed that the ‘function’ question must be construed more broadly than the related question of whether a trait is an adaptation *per se* (a trait shaped by natural selection to its current function). A trait can be useful, and increase survival and reproduction, without being an adaptation: an aversion to birth control might increase an individual's reproductive output, but is obviously not an adaptation *per se*. Thus, in following Tinbergen's rule we should clearly separate questions about what music is good for (seduction, social bonding, making a living, etc.) from the much harder questions about whether it is an adaptation for that or those purpose(s). Furthermore, questions of phylogeny (when did some trait evolve) are just as important as the ‘why’ question of function (see below).

Although Tinbergen's four questions provide excellent coverage for many biological traits, there is one domain of causation that he apparently overlooked: the domain of *cultural change* over historical time. This is a class of causal explanations spanning, in temporal terms, between the domain of individual ontogeny and species phylogeny (and is sometimes confusingly referred to as ‘evolution’, as in ‘the evolution of English’ or ‘the evolution of rap music’). This level of explanation is linked to, but independent of, both ontogeny and phylogeny. The issue is clearly exemplified by historical change in human language: there are many interesting questions concerning language where neither ontogenetic nor phylogenetic answers would be fully satisfying. For example, we might ask why an English-speaking child tends to place the verb second in declarative sentences, after the subject and before the object (so-called SVO basic word order). An ontogenetic answer would be ‘because that's what her parents do’ and an ultimate answer ‘because her ancestors evolved the capacity to learn language’. Although neither is incorrect, these answers leave out a crucial intervening level of explanation, concerning English *as a language*. English, like all languages, changes gradually over multiple generations by virtue of being learned anew, with minor variations, by each child. This iterated process of learning leads to a novel cultural level of explanation, sometimes termed ‘glossogeny’ [28,29], that can be studied productively in computational models and/or laboratory experiments [30,31]. The glossogenetic answer to the SVO question is complex, and part of the general domain of historical linguistics (it involves such factors as basic word order in Proto-Germanic and the overlay of French after the Norman Conquest [32]).

Returning to music, we know much less about the cultural evolution of most musical genres and idioms over time than we do about historical change in language. Nonetheless, it seems safe to assume that many interesting musical phenomena will find insightful explanations at this level (cf. Merker *et al.* [33]). One nice example concerns the dual origins of much contemporary popular music in the fusion of the harmonic and melodic traditions of Western Europe with the syncopated, polyrhythmic traditions of West Africa, brought together historically by slavery in the Americas [34–36].

Summarizing, Tinbergen's rule exhorts us to investigate each meaningful level of biological causation, and not to prioritize any single level over the others. Ultimately, bio-musicology will seek an understanding of musicality from mechanistic, ontogenetic, phylogenetic, functional and cultural viewpoints. Even if any particular researcher chooses to focus, for reasons of personal interest or empirical expedience, on some subset of these questions, the field as a whole should seek answers to all of them.

### (c) The comparative principle: adopt a comparative approach, embracing both homology and analogy

The first two principles urge us to isolate and analyse subcomponents of musicality and to approach their biology and evolution from a multifaceted Tinbergian viewpoint. The third and fourth principles concern our *sources of data* in this endeavour.

The third principle—‘be broadly comparative!’—urges a biologically comparative approach, involving the study of behavioural capacities resembling or related to components of human musicality in a wide range of non-human animal species. This principle is of course a question familiar to most biologists, but remains contentious in musicology or psychology. ‘Broad’ in this context means that we should not limit our biological investigations to close relatives of humans (e.g. non-human primates) but should rather investigate *any* species exhibiting traits relevant to human musicality.

The capacity for complex vocal learning nicely illustrates the need for broad comparison. This capacity underlies our ability to learn and share new sung melodies, and is shared with a diverse set of bird and mammal species (the current species count includes songbirds, parrots, cetaceans, hummingbirds, seals, bats and elephants) but is *not* found in any non-human primate. By contrast, the human propensity to generate percussive sounds via limb movements (‘drumming’) is shared both with our nearest primate relatives (gorillas and chimpanzees) and also with woodpeckers, kangaroo rats and palm cockatoos [26]. Similarly, chorusing and turn-taking among two or more individuals, a ‘design feature’ of human musicality, is seen in various forms in duetting primate and bird pairs and in a wide diversity of frog and insect species [37–40]. Thus, depending upon the specific component under investigation, the set of animal species that are relevant may be quite different.

Similar traits can be found in different species for several different reasons, and these are given specific names by biologists. In one type, termed ‘homology’, a shared trait is present in related species because a common ancestor of those species possessed the trait. Thus, all birds have feathers because the last common ancestor (LCA) of all living birds had feathers. All living mammal species produce milk to suckle their young, because their LCA produced milk. These are canonical examples of homology. A second class of shared traits are those that evolved independently or ‘convergently’ in two different clades; such traits can be termed *analogies* (the more technical biological term ‘homoplasy’ refers to all shared traits that are *not* homologies, and includes analogy as a special case). Canonical examples of analogy include the independent evolution of wing from forelimbs in birds and bats, or the evolution of bipedalism (walking on two feet) in humans and birds. Neither wings nor bipedalism were present in the quadrupedal reptilian LCA of mammals and birds, but instead evolved convergently in each of these clades.

Analogous and homologous traits play different roles in biology, but both are important. Homologous traits are those that are used in classification and taxonomy (for this purpose, analogous traits are just a nuisance variable). More relevant to bio-musicology, homologies often allow us to make inferences about traits that were present in an ancestral species, because a set of homologous traits in a particular clade are by definition inherited by descent from a common ancestor of that clade. Often, particularly for behavioural or cognitive capacities, homology-based phylogenetic inference is the *only* means we have of reconstructing these extinct ancestors, because behavioural traits typically leave no fossils (fossil footprints providing one exception). For example, although we will probably never find a fossilized Cretaceous stem mammal in the act of suckling her young, we can nonetheless infer, with great confidence, that the ancestral mammal did so from the fact that all living descendants of this species still do. Thus, a careful analysis of living species, combined with comparative inference, provides a sort of ‘evolutionary time machine’ to reconstruct the behaviour and physiology of long-extinct species.

Analogous traits serve a different and complementary purpose: they provide a means for testing hypotheses using multiple independent data points. Although all of the more than 5000 existing species of mammals suckle their young, this ability derives from their evolutionary origin at the base of the clade, and thus statistically constitutes a *single* data point (not 5000). By contrast, convergently evolved traits are by definition independent evolutionary events, and each clade independently possessing a trait therefore represents an independent data point. Only a set of convergently evolved traits provides an adequate database for statistically valid tests of evolutionary hypotheses. This point is often ignored, even by biologists discussing music evolution (e.g. [23]). Fortunately, for many cases of convergent evolution, such as bipedalism or vocal learning, a trait has evolved independently enough times to provide a rich source of evidence to test hypotheses concerning both evolution and mechanistic function. Thus, for example, we can test mechanistic hypotheses about the requirements of vocal learning by examining its neural correlates in the many species that have evolved this ability convergently (cf. [41]). Similarly, we can test functional hypotheses about why the capacity for vocal synchrony or antiphony is adaptive by examining the many bird, mammal, frog and insect species that have convergently evolved this ability [40].

While the conceptual distinction between homology and analogy is clear, recent discoveries in genetics and neuroscience suggest that in some cases a trait can be *both* homologous and analogous, depending on the level of explanation. For example, while eye and wings have both evolved independently in insects and vertebrates, it turns out that they rely in both cases on an identical set of genes and developmental pathways. This situation of convergent evolution ‘taking the same path twice’ has been termed *deep homology* [42,43]. This appears to be the situation for the capacity for complex vocal learning, which has evolved convergently and independently many times (reviewed in [41]). Nonetheless, comparisons of birds and humans reveal that the same genes (e.g. *FOXP2*) play a role in vocal learning in both groups [44], and that homologous neural mechanisms have been independently harnessed into vocal learning systems in birds and humans [45]. In both cases, there appears to be a deep mechanistic homology between birdsong and human vocal learning, despite their independent evolutionary origins (cf. [46–48]).

In summary, principle three exhorts bio-musicologists to adopt a broad comparative approach to any specific capability proposed as relevant to musicality. While it is important to distinguish homologous traits from those that convergently evolved, there is no justification for ignoring the latter (e.g. [23]), because both serve useful roles in comparative biology.

### (d) The ecological principle: seek broad ecological validity including popular styles, eschewing elitism

Like the previous one, this principle is also broadly comparative but this time involves comparisons *within* our species. According to this populist ‘ecological’ principle, bio-musicologists should seek to understand all manifestations of human musicality, from simple nursery tunes or singing in the shower, to expert bowmanship on a Stradivarius or the complex polyrhythmic improvisations of a Ghanaian master drummer. This principle is familiar to ethnomusicologists but not as widely appreciated by researchers in music cognition or neuroscience, where a focus on the Western ‘high art’ canon remains evident. Although it is of course important to understand highly developed musical forms, performed by elite musicians, this should not lead us to neglect more basic and widespread expressions of musicality.

The ecological principle is particularly important when addressing questions about the functional, adaptive relevance of music in our species (cf. [49]). It makes little sense to ask about the evolutionary ‘survival value’ of writing or performing a modern orchestral piece, but it is not unreasonable to ask about the potential adaptive value of a mother singing to her child, or of a tribal group singing and dancing together. Much of traditional musicology adopts an implicitly elitist attitude, where the proper object of study is ‘high’ art, composed and performed by a musical elite. Sometimes such elitism is explicit: a textbook intended to introduce students to music and art appreciation states that art ‘which aims merely to amuse and to provide a pleasant diversion … has little or no lasting quality’. In particular, the authors state that, ‘art which caters to the masses … is of little aesthetic value and will not be considered’. [50, p. 1]. But if we ever hope to understand the shared biological basis of music, it is *precisely* popular music style (e.g. dance music) that will be most relevant, along with behaviours such as a mother singing lullabies in order to soothe her infant: one of the functions of song for which the empirical data is most convincing [51,52]. An elitist attitude can thus lead us to overlook aspects of musicality that are centrally relevant biologically.

Equally important are the cognitive abilities of self-avowed ‘non-musicians’. One of the most fundamental findings in the last two decades of music cognition research is that untrained listeners, including those who claim they know nothing about music, exhibit sophisticated perceptual and cognitive abilities implying rich implicit understanding of musical principles (cf. [53–55]). In many cases such capabilities are already present in infants and children as well [12,13,56]. Any scientific exploration of the biological basis of human musicality should therefore take a broad view of musicality, across ages and over multiple levels of skill or training. This is not to say that musical expertise should be ignored as an explanatory factor: contrasts between highly skilled musicians and untrained listeners can provide a valuable source of data to help address mechanistic and developmental questions. But a focus *only* on the musical elite may often prove fundamentally misleading.

A third important facet of this principle concerns the diverse functions of music in human societies, with different functions shaping the expression of musicality in fundamental ways. For example, music created for dancers will typically have a clear and steady rhythm, as will most music intended for simultaneous performance by multiple individuals [35]. In both cases, a steady and explicit rhythmic framework is a crucial asset in group synchronization. By contrast, music for solo performance that is intended to express sorrow will develop under very different constraints, and may show no clear isochronic beat at all [57–59]. Only by studying the multiple contexts in which human musicality is expressed can we begin to make meaningful generalizations about the overall function(s) of music (cf. [22]).

Principle four thus states that, in order to obtain an ecologically valid overview of human musicality, we need to take a broad, populist and non-elitist viewpoint about what ‘counts’ as music. While high art music of many cultures is certainly relevant in this endeavour (including Western orchestral symphonies, Ghanaian agbekor improvisations, North Indian ragas or Balinese gamelan), so are folk music, nursery tunes, working chants and even whistling while you work or singing in the shower. Dance music in particular should be embraced as one of the core universal behavioural contexts for human music, and dance itself accepted as a component of human musicality.

## 3. Four core components of musicality

To illustrate how the four principles above interact constructively, let us return to the question raised by the multicomponent principle: ‘What *are* the biologically relevant components underlying human musicality?’ One first attempt at answering this question might combine the comparative and ecological principles to ask what functions music performs in human societies, and to what extent we can identify mechanisms underlying those functions in non-human animals. This approach leads us to recognize at least four subcomponents of music, as described below.

### (a) Song: complex, learned vocalizations

Let us start with *song*, one of the few aspects of human musicality that virtually all commentators agree is universally found in all human cultures [2,60–62]. Perhaps the most obvious fact about human song is that it varies considerably between cultures, and much less so *within* cultures (e.g. [3]). That is, each culture has both a shared, open-ended repertoire of specific songs, and culturally specific styles or idioms that encompass multiple songs. This situation is only possible when songs can be learned—so a child or newcomer can absorb the song repertoire of its community—and new songs can be generated within the style. This aspect of human song therefore entails the capacity for complex vocal learning, where novel sounds can be internalized and reproduced (cf. Merker *et al.* [33]). Having identified this particular ‘design feature’ of human singing, we can now ask which non-human species share this feature (cf. [26]). As already noted above, many different species have independently evolved the capacity for complex vocal learning, providing a rich comparative database for understanding singing from the multiple perspectives of Tinbergen's rule.

The criterion of vocal learning also provides a non-arbitrary way in which we can decide whether an animal species has ‘song’ or not. Past commentators have typically used implicit, intuitive criteria to decide this issue. For example, Hauser & McDermott [63] suggest that three animal groups have ‘animal song’: songbirds, humpback whales and gibbons. By contrast, Geissman's [64] review of gibbon song suggests that song exists in four primate groups: gibbons, tarsiers, indri and langurs, a list that has been further propagated uncritically in the literature (e.g. [27]). These papers provide no definition of animal song, nor any justification for their different lists. By contrast, Haimoff [38] does offer a definition of song—animal sounds that ‘are for the most part pure in tone and musical in nature’ (p. 53)—and then nominates the same four primate clades listed by Geissman as *duet* singers. But lacking wide agreement about what ‘musical in nature’ means, this definition is not very helpful. It remains entirely unclear why none of these authors consider the complex, multi-note pant-hoot displays of chimpanzees, with their marked crescendi and drummed finale [65], or the tonal ‘combination long calls’ of cotton-top tamarins [66], or a host of other primate vocalizations to be ‘song’. Explicitly stating without justification that chimpanzees do *not* have song, Hauser & McDermott [63] go on to conclude that ‘animal song thus likely has little to do with human music’ (p. 667). But here the attempt at a comparative analysis has misfired at the first step: without any objective and non-circular criteria to define ‘song’ we cannot even objectively state what species have, or lack, song—much less evaluate its potential relevance to human music.

By contrast, if we identify vocal learning as a core defining feature of human, bird and whale ‘singing’, we obtain a clear and unambiguous criterion that allows us to adopt a meaningful comparative perspective [26]. This is why I have previously argued that a musically relevant definition of song is ‘complex, learned vocalization’, irrespective of tonality or any aesthetic qualities these complex vocal displays might possess to our ears. While the aesthetic virtues of the rough and sputtering underwater vocal displays of a harbour seal remain a matter of taste [67,68], it is clear that this species *does* have a capacity for vocal learning [69]. Furthermore, dialectal variations among populations of harbour seals and some other pinniped species suggest that this ability allows seals to learn locale-specific vocal displays [70–72]. By my definition, the displays of songbirds, parrots, whales or seals can be termed ‘animal song’, and considered analogous to human singing, but the displays of chimpanzees, gibbons, indri and other non-human primates cannot, because these primate displays, though complex and beautiful, are not learned. I do not object if those scientists studying the haunting choruses of the indri or the territorial displays of gibbon pairs continue to use the traditional term ‘songs’ for these unlearned vocalizations. For that matter, people can freely apply the term to frog, cricket or fish ‘songs’, or even ‘the song of the forest’. But in the scientific context of comparisons with music, I think that such colloquial usage, without any clear and non-arbitrary guidelines or objective justification, is deeply misleading.

### (b) Instrumental music: percussion and drumming

Of course, humans do not express our musicality solely by singing: virtually all human cultures also have instrumental musical traditions. By ‘instrumental music’, I simply mean the creation of communicative acoustic signals through non-vocal means. This broad definition includes the highly developed harmonic string and wind ensembles typical across Eurasia, the timbrally complex and more percussive gamelan tradition of Southeast Asia, and the complex polyrhythmic drum ensembles of sub-Saharan Africa. The earliest unequivocal archaeological evidence for musicality in our species is represented by instruments: numerous bone flutes have been found throughout Eurasia that document sophisticated human music-making at least 40 000 years ago [73–76] and other putative musical instruments are also known (cf. [49]). However, while ‘aereophones’ are certainly common in human music across the world, they are not universal. The one form of instrumental music that is (very nearly) universal is the use of percussive instruments: ideophones and drums [60,61]. I will thus focus on percussive drumming here, as a second core component of human musicality.

From a biological comparative viewpoint, there are many interesting parallels with human drumming in nature. It is much harder to find parallels with other instrument types, but spiders plucking and vibrating their webs might be considered as a distant analogue of stringed instruments [77]. Defining percussive drumming as the production of structured communicative acoustic signals by striking objects with limbs, other body parts, or other objects, we find several instances in other species. Starting with analogues, woodpeckers (bird family Picidae) produce displays by striking hollow trees with the bill [78,79], and multiple species of desert rodents produce audible and far-carrying seismic signals by pounding the ground with their feet [80]. Both of these examples help to clarify the distinction between ‘structured communicative sounds’ and sounds that are an incidental by-product of other behaviours. Any organism generates footfall sounds when it locomotes, but rodents' communicative drumming displays are produced without locomoting, in particular locations (often within their burrow), and in specific contexts (territorial displays and/or predator alarms [80]). Similarly, woodpeckers make incidental sounds when foraging for wood-boring larvae, but during their drumming displays they seek out particularly resonant trees (or in urban environments, other resonant objects such as hollow metal containers on poles). Again these displays are made in particular contexts, including territorial defence and advertisement, and often are both identifiable as to species and bear individual-specific ‘signatures’ [78,81]. Thus, these displays show every sign of having evolved for the purpose of influencing others, and thus constitute animal signals by most definitions (e.g. [82,83]).

Turning to primates, many ape and monkey species generate non-vocal sounds as part of communicative displays (e.g. branch shaking, or cage rattling in captivity [84]). Orangutans have been reported to modify the frequency content of their vocal displays using leaves placed in front of the mouth, an example of ‘tool use’ which blurs the line between vocal and instrumental displays [85]. But the most striking example of instrumental behaviours in primates comes from the drumming behaviour of our nearest living relatives, the African great apes (gorillas, chimpanzees and bonobos). While still little studied, these behaviours include drumming on resonant objects with the feet or hands, typical of chimpanzees, and drumming with the hands on the chest or other body parts, by gorillas [26,86–88]. Clapping by striking the hands together is also commonly seen in all three species in captivity, and has been observed in the wild in chimpanzees and gorillas [89,90]. There is strong evidence that such percussive drumming is part of the evolved behavioural repertoire of African great apes: it is consistently observed in both wild and captive animals, exhibited in particular contexts (displays and play), and when it involves objects, they are often particularly resonant objects apparently sought out for their acoustic properties [86]. Drumming thus represents not just a universal *human* behaviour, but also one that we share with our nearest living relatives. Drumming is thus a clear candidate for a homologous behavioural component of the entire African great ape clade, of which humans are one member. Applying the phylogenetic logic of the comparative principle, this suggests that drumming evolved in the LCA of gorillas, chimpanzees and humans, who lived roughly seven or eight million years ago in the forests of Africa [91].

Even a brief survey of animal instrumental music would be incomplete without mentioning the palm cockatoo, *Probosciger aterrimus*, a large parrot species living in Australia and New Guinea. Male palm cockatoos use a detached stick, held in the foot, to strike on resonant hollow branches as part of their courtship displays [92,93]. They are also occasionally seen to drum with the clenched foot alone, but much more quietly, suggesting that this sole animal example of *tool-assisted* drumming may have evolved from a limb-based drumming comparable to that seen in chimpanzees. This provides an interesting parallel to human drumming, where the hand drumming that we share with other apes is often augmented by drumming with tools like sticks or mallets.

In summary, drumming appears to constitute another core component of human musicality with clear animal analogues. In the case of the African great apes percussive drumming appears to constitute a *homologous* trait, suggesting that this component of human musicality evolved in the LCA of humans, gorillas and chimpanzees more than seven million years ago.

### (c) Social synchronization: entrainment, duets and choruses

A third core component of human musicality is our capacity to synchronize our musical behaviours with others. This may be by performing the same action at the same time (e.g. clapping or chanting in unison—synchronization *sensu strictu*) or various more complex forms of entrainment such as antiphony or the complex interlocking patterns of an agbekor drum ensemble. Although solo music, performed by a single individual, is not uncommon, music performed in groups is a far more typical expression of human musicality. This is again a universal behaviour seen in at least some of the music of all human cultures [60], and such coordinated group displays also find important parallels in the animal world.

Social synchronization requires individual capacity for synchronization to some external time-giver. The most sophisticated form of synchronization involves beat-based predictive timing, where an internal beat is tuned to the frequency and phase of an isochronous time-giver, allowing perfect 0° phase alignment. This capacity to extract an isochronic beat and synchronize to it is termed ‘beat perception and synchronization’ or BPS [94]. Although the majority of research in both humans and animals studies BPS to either a metronome or recorded musical stimuli [95,96], human rhythmic abilities obviously did not arise to allow people to synchronize to metronomes, but rather to the actions of other humans, in groups. Thus, by the ecological principle, the concept of ‘mutual entrainment’ among two or more individuals should be the ability of central interest, rather than BPS to a mechanical timekeeper.

Despite a long tradition of suggesting that BPS is uniquely human, recent findings clearly document this ability in several species, including many parrot species [97–99] and more recently a California sea lion *Zalophus californianus* [100]. By contrast, the evidence for BPS in non-human primates remains weak, with partial BPS by a single chimpanzee and not others [101]. Thus, the existing literature suggests a lack of BPS abilities in other non-human primates (see Merchant *et al*. [10], and [102–104]). Thus, while human BPS clearly finds analogues in the animal kingdom, it is too early to say whether homologous behaviours exist in our primate relatives. But again this aspect of human musicality provides ample scope for further comparative investigation (cf. [105]).

Synchronization in larger groups—‘chorusing’—is also very broadly observed in a wide variety of non-human species, including frogs and crickets in the acoustic domain and fireflies and fiddler crabs in the visual domain (for reviews see [37,40]). In some cases choruses involve BPS. For example, in certain firefly species, all individuals in a tree synchronize their flashing to produce one of the most impressive visual displays in the animal kingdom [106–108]. These cases all represent convergently evolved analogues of BPS, and thus provide ideal data for testing evolutionary hypotheses about why such synchronization capacities might evolve, along with mechanistic hypotheses about the minimal neural requirements supporting these capacities. Although frog, cricket and firefly examples are often neglected in discussions of music evolution, presumably because they are limited to a particular signalling dimension and a narrow range of frequencies, there are some species which show a flexibility and range of behaviours that is musically interesting. For example the chirps of tropical *Mecapoda* katydids are typically synchronized (predictively entrained at 0° phase) but under certain circumstances can also alternate (180° phase) or show more complex entrainment patterns, and over a broad range of tempos (chirp periods from 1.5 to 3 s, [109]). Thus, even very small brains are capable of generating an interesting variety of ensemble behaviours in chorusing animals—raising the fascinating question of why such behaviours are rare in so-called ‘higher’ vertebrates like birds and mammals.

Other less demanding forms of temporal coordination also exist, but these forms of multiindividual coordination have been less researched and discussed (even in humans). These include turn-taking or call-and-response pattern, and can be accomplished using reactive rather than predictive mechanisms (e.g. ‘don't call until your partner has finished’). Again such abilities find many parallels in the animal world. The most widespread examples are found in duetting birds or primates, typically between the male and female of a mated pair. Over 90% of bird species form (socially) monogamous pairs, exhibiting joint parental care and often joint territory defence. It is thus unsurprising that coordinated duetting is common, and better-studied, in birds than in most other groups [39,110–114]. Avian duetting, like female song more generally, is more common in tropical non-migratory species than in temperate climates [115,116], and the ancestral state of songbirds may have included both male and female song [117].

Duets have also evolved convergently in at least four monogamous primate species [38]. Typically in duets, the male and female parts are temporally coordinated and interlock antiphonally, and this temporal coordination requires some learning by the pair members to become fluent. However, there is no evidence for vocal learning of the calls themselves, which (especially for gibbons) are innately determined [64]. Gibbon duets probably rely on reaction-based turn-taking and do not appear to require predictive BPS mechanisms, but this remains an under-studied area.

Although it is rare, some bird species also show a mixture between duetting and chorusing. The plain-tailed wren (*Thryothorus* = *Pheugopedius euophrys*) is a member of a clade in which all species show duetting [118], but unique to this species, the birds often live in larger mixed-sex groups that sing together. During territorial song displays, the female and male parts interlock antiphonally in the normal way, but multiple females sing the female part in perfect synchrony, while the males also combine their parts synchronously, with remarkably exact timing [119]. In general, duetting and chorusing provide a rich set of analogues to human ensemble behaviour, allowing both the evolution and mechanistic basis of such behaviours to be analysed using the comparative method.

### (d) Dance: a core component of musicality

I conclude with a component of human musicality that has been unjustly neglected in most discussions of the cognition and neuroscience of music: our capacity to dance. Although English and many other European languages distinguish ‘music’ from ‘dance’, this distinction is not made in many other languages, where music and dance are considered to together comprise a distinctive mode of human interaction (cf. [24,27,61]). A close linkage between music and dance is also evident in most European music outside the concert hall, and although dance may be *distinguished* from music, it is almost always *accompanied* by it. Furthermore, so much of human music is created for the express purpose of dancing that, in the development of many musical styles (e.g. waltz or swing), dance and music have undoubtedly influenced each other deeply [120]. Finally, dancers make use of the synchronization abilities just discussed, to synchronize with the music and/or with other dancers. Thus I nominate dance as another core component of human musicality.

It is not trivial to define dance, and probably foolhardy to seek a definition that clearly distinguishes it from other aspects of musicality. Again starting from the comparative viewpoint, there are a vast array of visual displays among animals, from claw-waving in crabs to begging gestures in apes, many of which are probably not relevant to human musicality. With such comparisons in mind, I will provisionally define dance as ‘complex, communicative body movements, typically produced as optional accompaniments to a multimodal display that includes sound production’. This definition picks out the core of most human dancing without attempting to distinguish it strictly from drumming: by this definition tap dancing constitutes *both* dancing and drumming simultaneously. Chimpanzee drumming is typically the culmination of a multimodal display that includes both vocal elements (pant-hoot) and a swaggering and rushing about; I am happy to consider this a form of dancing. By my definition, the expressive movements often made by instrumentalists as they play, over and above those necessary to produce the sounds, would also be classified as dancing, as would head bobbing, foot tapping or hand movements made by listeners in synchrony with music. While I am aware that pantomime, or some ‘high art’ dance, may be performed silently, I do not find such rare exceptions particularly troublesome (any more than John Cage's famous 4′33″—a ‘musical’ piece involving no sound—should constitute a central problem in defining music). If we seek comparisons that help fuel scientific, biologically oriented research, we should seek useful generalizations rather than perfect definitions.

When searching for animal analogues of dance, it is important to note that multimodal signalling is a ubiquitous aspect of advertisement displays in animals, and probably represents the rule rather than the exception (cf. [121–123]). For example, many frogs have air sacs which are inflated when the frog calls. In some species, these sacs are decorated in various ways and thus serve as simultaneous visual displays; studies with robot frogs demonstrate that both components of these multimodal displays are attended to by other frogs [124]. But because vocal sac inflation is a mechanically necessary part of the vocal display, rather than an *accompaniment* to that display, I would not consider this to be ‘dance’. However, a frog that, in addition, waves its feet while calling *would* be dancing by my definition (cf. [125,126]). The clearest potential analogues of human dancing are seen in the elaborate and stereotyped visual/vocal displays seen during courtship in many bird species, such as birds of paradise, ducks, grebes, cranes and many other species. In the case of cranes, for example, courtship is a protracted affair that includes elaborate, synchronized species-typical body and neck movement in addition to the pairs' synchronized calling behaviour [127,128]. These are traditionally, and I think rightly, referred to as ‘dance’. Other multimodal displays exist that seem intuitively to be dance-like, e.g. the ‘stiff walking’ seen during aggressive display in red deer, accompanied by roaring, or the ‘swaggering’ gait, with full piloerection, often seen during pant-hoot displays in chimpanzees, are quite difficult to quantify, but deserve further study.

Although animal ‘dancing’ behaviours remain relatively unexplored, particularly in the context of bio-musicology, I suggest that accepting dance as a core component of human musicality will open the door to further fruitful comparisons, uncovering both analogues and possible homologues in other species. More generally, I suggest that bio-musicology will profit greatly by explicitly incorporating dance into discussions of the biology and evolution of human music. It is time to recognize dance as a full peer of song or drumming in human expressions of musicality.

## 4. Conclusion

In closing, I re-emphasize that both the principles and components discussed in this essay are offered as starting points. I fully expect, and hope, that as the field of bio-musicology progresses more principles will be developed, or the ones presented here augmented and refined. In particular, the four-component breakdown I have given above is just one way to ‘slice the pie’ of musicality, developed specifically for the purposes of fruitful comparisons among species. Two other important multicomponent analyses include the search for musical universals of various types (see below), and the attempt to break music into ‘design features’ which allow a matrix of comparisons between music and other human cognitive features (such as language or architecture) and with other animal communication systems, following Hockett [129]. Hockett's list of design features of language provided an important starting point for subsequent research in animal communication, and elsewhere I have offered a list of *musical* design features extending his [26,130]. My list includes some features that are shared with language (such as generativity and complexity) as well as features that differentiate most music from language (such as the use of discrete pitches, or of isochronic rhythms), but shorter lists of musical design features have also been proposed [131]. The ‘design feature’ approach focuses on characteristics of *music* rather than on the cognitive abilities making up *musicality*, but may be preferable in cases where we have empirical access only to surface behaviours. There is thus plenty of room for expansion and exploration of this feature-based approach to analysing musicality into component parts.

Another important alternative approach to analysing the components underlying musicality is much older, and much more controversial: the search for musical universals. This was a core desideratum of the first wave of comparative musicologists, centred in Germany between the wars [132–134]. Unfortunately, with a few exceptions [3,4,135–137], the search for universal principles or traits of music was abandoned after the breakup of this group of researchers by the Nazis. Indeed, in post-war ethnomusicology the very notion of musical universals became somewhat taboo and, in line with prevailing attitudes concerning culture more generally, music was seen as a system free to vary with virtually no constraints (cf. [61,138,139]). But the steady increase in the scientific study of music, particularly music neuroscience and music cognition, has led a few brave scholars to reopen this search [60,61]. This empirical quest to derive broad generalizations about human musicality is clearly an important component of bio-musicology that has been neglected for too long.

Bio-musicologists may learn some important lessons from the long-running discussions of language universals in linguistics (cf. [140]). The earliest modern attempts to empirically analyse language universals were led by comparative linguist Joseph Greenberg [141], who clearly distinguished between truly universal traits (e.g. ‘all languages have both nouns and verbs’), statistical universals (‘most languages have trait *x*’) and implicational universals. Implicational universals are the most interesting: they take the form ‘if a language has trait *x*, it will also have trait *y*’, and again may be truly universal or just strong statistical generalizations. I know of few discussions of this type of universals concerning musicality, but Temperley [35] has offered a fascinating set of candidate topics for this type of implicational generalization in music. For example, Temperley suggests a trade-off between syncopation and rubato (free expressive variation in tempo) as a musical style evolves, arguing convincingly that syncopation only works well in the context of a relatively strict isochronic beat (because otherwise time-shifts intended as syncopations become indistinguishable from expressive temporal dynamics).

After Greenberg, the discussion of language universals became more heated when Noam Chomsky introduced his controversial concept of ‘Universal Grammar’ or UG, adapting an old seventeenth century term to a new purpose [142]. The debate this concept sparked has often been unproductive, mainly due to the frequent conflation of UG (the capacity to acquire language) with superficial traits found in all human languages (Greenberg's ‘true universals’). Since true universals are unusual, their rarity has frequently been claimed to disprove the concept of UG itself (e.g. [143,144]), despite the fact that Chomsky stressed his focus on ‘deep-seated regularities’—very general aspects of the *capacity* to acquire and use language, such as its creative aspect—and not on traits found in all human languages [142, pp. 5–7]. Bio-musicology, and musicology more generally, will do well to learn from this history of linguistic debate over language universals, lest we be doomed to repeat it. The key point is that some particular capacity may well be a universal trait of human *musicality* (available as part of the cognitive toolkit of any normal human) without being expressed in all musical styles or found in all human cultures. For example, humans around the world have a capacity to entrain our movements to musical rhythms, but we do not express this ability with every form of music. Indeed, for some non-isochronic ‘free’ rhythms this would be both difficult and culturally inappropriate [57]. But there is no conflict in claiming that synchronization to isochronic rhythms is a universal human capacity, and observing that it is not observed in all musical pieces, styles or cultures (cf. [60]). A similar point could be made, *mutatis mutandis*, concerning melodic grouping or harmonic ‘syntax’.

In conclusion, while the principles and components introduced here are preliminary and by no means exhaust the store, I hope to have shown how adopting *some* explicit breakdown and then proceeding to study each component comparatively opens the door to rich and exciting sources of data to help understand the biology and evolution of music. Asking monolithic questions like ‘When did music evolve?’ is unlikely to be productive, but questions like ‘When did our propensity to drum with our limbs evolve?’ can already be tentatively answered (around eight million years ago, see above).

Similarly a question like ‘*Why* did music evolve?’ must immediately grapple with the broad range of uses to which music is put in human cultures. By contrast, the question ‘Why did the human capacity to *entrain* evolve?’ is one that we can begin to answer by employing the comparative approach, given the many species that have convergently evolved this ability. Again, the exact breakdown is likely to remain a matter of debate for the foreseeable future, and will be dependent on the specific problem being addressed. But I suggest that the need for *some* breakdown is a core prerequisite for future progress in this fascinating field of research.
